# Pulling at the Roots: Broadening Our Perspective on Mental Disorder Aetiology

**DOI:** 10.1017/gmh.2014.5

**Published:** 2014-09-15

**Authors:** Soraya Seedat

At a global level, our understanding of mental illness has unparalleled potential for breakthroughs given recent advances in genomics, neuroimaging, and the cognitive, affective, and social neurosciences. The emergence of high-throughput and low-cost genomic technologies, and the convergence of a number of new genomic approaches with systems biology approaches and with neuroimaging data, in the context of large-scale studies, has paved the way for more computationally based neuroscience that will arguably enable us to bridge the knowledge gap between causation and psychopathology. Major initiatives such as the European Union-funded Human Brain project and the USA-funded BRAIN (Brain Research through Advancing Innovative Neurotechnologies) project are focusing efforts on multilevel integration of brain neuronal networks and connections, using intensive computing, in an endeavour to create the next generation of brain tools that will inform a deeper understanding of the brain (Kandel *et al.* 2013). In the future, these tools are likely to have profound therapeutic and preventive applications.

With these promising advances there has, paradoxically, been a fair amount of frustration voiced in the Global Mental Health Community by researchers and clinicians alike. These frustrations concern the application and potential uses of such tools that extend to the: (i) relatively slow pace of progress in completely unravelling the complex aetiology of mental disorders; (ii) absence of reproducible, disease-specific biomarkers; (iii) wide chasm between clinical diagnosis and molecular and brain neuroscience; (iv) relatively poor integration between brain neuroscience and social and cultural variables; and (v) lack of recent treatment breakthroughs against the backdrop of a high global burden of psychiatric morbidity (Krystal & State, 2014).

But in addition, these frustrations reflect the distance such tools are from much of the developing world and from much of the social, psychological, and cultural aetiologies of which they are a part and whose understanding has also been impoverished by being primarily studied in only some populations and in only certain parts of the world. For mental health researchers and clinicians working in low- and middle-income countries, scientific discovery and innovation is further hampered by lack of access to key technological platforms (e.g. epigenomics, proteomics, metabolomics, and neuroimaging facilities) and limited armamentarium of effective pharmacological, psychological, and social treatments. Nonetheless they have the potential to uniquely add to a global understanding of aetiology by markedly expanding the populations, contexts, and opportunities for study.

Psychosocial models of risk and causality in mental illness are as important as, and complementary to, biological frameworks. At a global level these advances, therefore, underscore the need to pay even more attention to other factors, and other needs (e.g. socio-economic deprivation, race/ethnicity/cultural issues, migratory stress, food security, and economic growth) that may contribute to disparities in mental health care use and treatment outcomes (Chiao & Blizinsky, 2013). To this end, a comprehensive understanding of the complex experiences of patients we see in the clinic and in the community not only requires an understanding, and far broader participation in exploration, of the molecular, cellular, and systems level aberrations that give rise to illness, but also of the individual, family, social, cultural and environmental factors, and health care system issues, that may be at play. Longitudinal assessment of social, cultural, and economic determinants of the onset and maintenance of mental disorders, in different geographical settings, will be necessary in this regard. There have been a number of intriguing observations that warrant mention. For example, social stressors in the form of abusive and/or poverty-stricken environments haven been consistently documented to be potent risk factors for a wide range of mental illness. Abuse, in particular early abuse, and impoverishment are epidemiologically validated risk factors that have been shown to interact with genes and impact neural circuits. Of themselves they are able to modify, through epigenetic mechanisms, genes and pathways implicated in psychiatric disorders. Social environment and epigenetics can, in turn, alter neurodevelopmental trajectories during childhood and adolescence when the brain and biology of individuals are particularly vulnerable to change (Meyer-Lindenberg & Tost, 2012).

Investigation of aetiology, therefore, requires a truly interdisciplinary approach, but also a truly global approach, both geographically, and disciplinarily; in the case of trauma, for example, a marriage of neuroscience, epidemiology, child psychology, and sociology, and the wider frame of experiences and contexts where trauma happens that a truly global perspective can bring. Another example of an epidemiologically validated factor is urbanicity, which is known to increase the prevalence and incidence of a number of psychiatric disorders (e.g. schizophrenia and mood disorders). Urbanicity and city living have also been found to increase stress-reactive brain regions (viz. amygdala and anterior cingulate cortex), important for the regulation of affect, during the performance of cognitive tasks under conditions of social stress (Lederbogen *et al.* 2011). These brain findings were notably regionally and behaviourally specific, suggesting that there are distinct neural mechanisms that coincide with this urban environmental risk factor to underpin disease causation. Furthermore, novel psychiatric treatment discovery (drug and non-drug) hinges on a constitutive understanding of the aforementioned contributions to illness causation, and identifying the ‘chains of risk’ is key (Papachristou *et al.* 2013).

We would like to provide readers of *Global Mental Health* (*GMH*) with a wide-lens perspective on the global commonalities and differences in the range of emerging knowledge around the macro- and molecular-level determinants of psychiatric disorders. This is one of our aims and *GMH* seeks to present original data and reviews on the various forces (genes, environment, brain, behaviour, economic, sociopoiltical, and cultural forces) that contribute to risk and resilience in psychopathology in different parts of the world, and over the life course. A more fine-grained understanding of how and why populations differ in their risk and resilience to psychiatric disorders is paramount.

Papers that focus on current controversies and challenges in the field, and that broaden the scope of geography and disciplines taking part in building this knowledge base, are encouraged. Additionally, as there is growing evidence that mental disorders are determinants for other non-communicable diseases (e.g. cardiovascular disease and type 2 diabetes), as well as for communicable ones (HIV and tuberculosis), more data are needed on mental disorders as independent risk factors for other diseases on a global level, the temporality of these associations, and their public health and policy impacts.

Reducing the burden of illness from mental disorders will be challenging and will require tackling the many ‘breaking blocks’ and ‘building blocks’ that underlie disease expression and health, both at an individual and population level. The field is poised to make rapid strides as we take a more nuanced and integrated view of the biological, environmental, behavioural, ecological, cultural, and social factors that will illuminate the aetiology of these disorders. Your contributions will be crucial to helping us get there!
